# The Clinical Efficacy and Safety of Gumiganghwal-Tang in Knee Osteoarthritis: A Phase II Randomized Double Blind Placebo Controlled Study

**DOI:** 10.1155/2018/3165125

**Published:** 2018-11-14

**Authors:** Sung Hae Chang, Yun-Kyung Song, Seong-Su Nah

**Affiliations:** ^1^Department of Internal Medicine, College of Medicine, Soonchunhyang University, Cheonan, Republic of Korea; ^2^Department of Korean Rehabilitation Medicine, Gachon University, Sungnam, Republic of Korea

## Abstract

**Background:**

Gumiganghwal-tang (GMGHT) is a traditional herbal medicine consisting of nine different herbs. GMGHT inhibits the mRNA expression and production of inflammatory cytokines tumor necrosis factor-*α* (TNF- *α*), interleukin-6 (IL-6), and TNF- *β* on lipopolysaccharide- (LPS-) stimulated peritoneal macrophages in a dose-dependent manner. It is empirically used for the treatment of inflammatory disease, but there are few reports of clinical trials that investigate its efficacy and safety. The current study aimed to investigate the clinical efficacy and safety of GMGHT in patients with knee osteoarthritis (OA).

**Methods:**

This was a multicenter, two-armed, double-blinded, randomized, placebo controlled study of GMGHT over 6 weeks. Eligible patients who fulfilled the American College of Rheumatology criteria for OA were randomized to receive either GMGHT or the placebo. Clinical assessments included measurement of knee pain and function using the Western Ontario and McMaster Universities Osteoarthritis Index (WOMAC), patient global assessment (PGA), and knee pain scores every 2 weeks.

**Results:**

A total of 128 patients were enrolled (91.4% female; mean age, 58.7 ± 8.1 years). At baseline, pain visual analogue score (VAS) was 67.2 ± 1.4, resp. 71.3 ± 1.6 (treatment, resp. placebo group, p=0.84), and total WOMAC score was 55.2 ± 1.6, resp. 55.6 ± 1.5 (p = 0.84). After 6 weeks, the pain VAS was 43.0 ± 2.5, resp. 61.6 ± 2.5 (p < 0.01) and the total WOMAC score was 34.1 ± 2.4, resp. 46.9 ± 1.8 (p < 0.01). No patients withdrew because of treatment emergent adverse events. Expected adverse events including dyspepsia, liver function abnormality, and lower extremity edema were comparable between both groups.

**Conclusions:**

Treatment with GMGHT resulted in significant improvement in pain, function, and global assessment, and it was generally safe and well tolerated in patients with OA.

## 1. Background

Osteoarthritis (OA) is a highly prevalent disease with substantial socioeconomical impact. OA commonly falls under the umbrella term of degenerative diseases that are associated with aging. Of note, the fact that nonsteroidal anti-inflammatory drugs (NSAIDs) improve pain more than paracetamol in certain patient groups of OA or that intra-articular triamcinolone injection showed superior results compared to intra-articular visco-supplementation for alleviating joint pain supports the pivotal role of inflammation in the pathogenesis of OA [1–3].

The pharmacological treatment for control of the active symptoms of osteoarthritis is mainly acetaminophen, NSAIDs, and tramadol [4, 5]. However, acetaminophen is less effective, and tramadol may cause dizziness or aggravated orthostatic hypotension especially in elderly patients. NSAIDs are known to be associated with increased cardiovascular and gastrointestinal risks [6–8]. Considering that up to 30% of patients with knee OA had 3 or more self-reported comorbid conditions, the current medical treatment options are not sufficient [9, 10]. Therefore, there have been many efforts to develop medications with adequate efficacy but without the associated side effects.

Gumiganghwal-tang (GMGHT) is composed of 9 plants, and the main components are* Angelicae pubescentis radix* and* Ledebouriellae radix*. It has been used to treat the common cold and arthralgia in Korean traditional medicine. The extract of* Angelicae pubescentis radix* showed analgesic and antiplatelet effects in an* in vivo* study and has been described as having a similar function to aspirin [11, 12]. GMGHT also has an analgesic effect comparable to piroxicam as demonstrated in an acetic acid induced writhing test. The GMGHT was able to ameliorate inflammation via inhibition of NF-kB resulting in the reduction of cytokine production* in vitro* [13, 14]. Several studies to evaluate toxicity of GMGHT revealed no-observed-adverse-effect-level and that GMGHT relieved pain via an anti-inflammatory effect. Considering the significant role of inflammation in OA, the aforementioned results suggest GMGHT may be another pharmacological treatment option for controlling OA. Therefore, in the current study, we evaluated the efficacy and safety of GMGHT in knee OA.

## 2. Material and Methods

### 2.1. Study Design

This study was conducted as a prospective, randomized, double-blinded, placebo controlled study at both Soonchunhyang University Cheonan Hospital and Gachon University Gil Oriental Medical Hospital from July 2010 to August 2011. The study was approved by the local ethical committees (Soonchunhyang University Cheonan Hospital: IRB-No. SCH 004, Gachon University Gil Oriental Medical Hospital). The study was performed in accordance with the protocol, and all subjects provided written informed consent.

Upon enrolment to the study, the participants had a washout period, at least 5 times the half-lives of concomitant NSAIDs or COX 2 inhibitors. The screening period including the washout period did not exceed a maximum of 2 weeks, and eligible participants were randomized for allocation to either the GMGHT group (study group) or placebo group (control group) in a 1:1 ratio. The researcher who was not involved in the trial had made a randomized list with sequential numbers, and patients were allocated serially. The participants and clinical investigator were blinded to the study interventions. Randomized patients visited the clinic every 2 weeks over the 6-week study period. Any concomitant medications for OA were prohibited during the study period except acetaminophen (Tyleno-ER R tablet 650 mg, Janssen Pharmaceuticals Inc., Titusville, New Jersey), which was allowed as a rescue medicine for pain relief if needed. The maximum dose of acetaminophen permitted was 3000 mg per day, which was refrained within 24 h of clinical assessment during the study.

### 2.2. Patients

Participants who met the following inclusion criteria were recruited to the trial: age 40 years or older, diagnosis of clinical symptomatic knee OA defined using the American College of Rheumatology (ACR) criteria for at least 3 months before screening, documented radiographic changes indicating OA of the knee (Kellgren-Lawrence grade greater than or equal to 2 at the time of screening), pain in at least one knee with a visual analogue scale pain score of ≥ 50 mm and < 80 mm ( 1-100 mm scale), pain requiring analgesia or NSAIDs at least 5 days or more than a week in the last month, not pregnant or lactating, use of effective contraception if participant is a woman of childbearing age, and a signed consent statement. Patients with congestive heart failure, uncontrolled hypertension, a history of stroke, renal artery stenosis, ischemic heart disease or renal failure, hypersensitivity or intolerance to NSAIDs/aspirin/COX-2 inhibitor, hemostatic dysfunction including concurrent warfarin treatment, and inability to provide informed consent, who were pregnant or lactating, were excluded. Patients were also excluded if they had concurrent disease resulting in secondary osteoarthritis such as a fracture, septic arthritis, or inflammatory arthritis, if they underwent orthopedic surgery within the 6 months prior to screening, or if they planned to have a surgical procedure during the study period.

### 2.3. Preparations

GMGHT is a mixture of nine traditional drugs as mentioned previously (Table 1). GMGHT is one of reimbursed traditional herbal medicine in Korea as anti-inflammatory, antipyretic, and analgesic drug. GMGHT is produced by several pharmaceutical companies. In the current study, a 43.9 g GMGHT granule and placebo was provided by Hanpoong Pharmaceuticals Inc. (additional drug descriptions can be found on the following webpage: http://www.hanpoong.co.kr/ab-goods-280-1001001003). The placebo granule is indistinguishable from GMGHT in its taste, smell, and color; its gradient is composed of excipients. The subjects took the GMGHT or placebo twice a day: one pack in the morning and one in the evening.

### 2.4. Outcome Measures

Outcome assessments were conducted at screening, baseline, 2, 4, and 6 weeks after randomization. Within 48 hours of each visit, the subject responded to the 5-point Likert-type version of the Western Ontario and McMaster Universities Osteoarthritis Index (WOMAC) questionnaires. The patient's VAS scale (0–100 mm) and pain assessment, and the physician's global disease assessment were also evaluated. Primary outcomes in this study were changes in the total WOMAC score. Secondary outcomes included the change in WOMAC, pain, function, and stiffness subscale score, the patient and physician's global assessment, and adverse drug reactions and adherence as measured by medication count and diary. The primary outcome of the current study was that 4-week GMGHT treatment would result in improvement of WOMAC scores.

### 2.5. Statistical Analysis

Assuming that GMGHT improves the WOMAC score by 10 mm and patient's global assessment by 0.5 from baseline, and assuming an *α* level of 0.05 (2-tailed), a power of 0.80, and a dropout rate of 20%, the sample size calculation revealed that 73 patients for each group were needed for enrolment. A per-protocol analysis was performed for the 127 patients who completed the 6-week study periods. A paired* t*-test was used to assess changes from baseline measurements for both the primary and secondary outcomes between each treatment group. An independent two-sample Student's* t*-test was used to compare differences between the treatment groups in the change from baseline for continuous outcome measures. P ≤ 0.05 was used as the level of significance for all analyses. All analyses were performed using PASW Statistics 18 (SPSS Inc., Chicago, IL, USA).

## 3. Results

**Figure 1:**
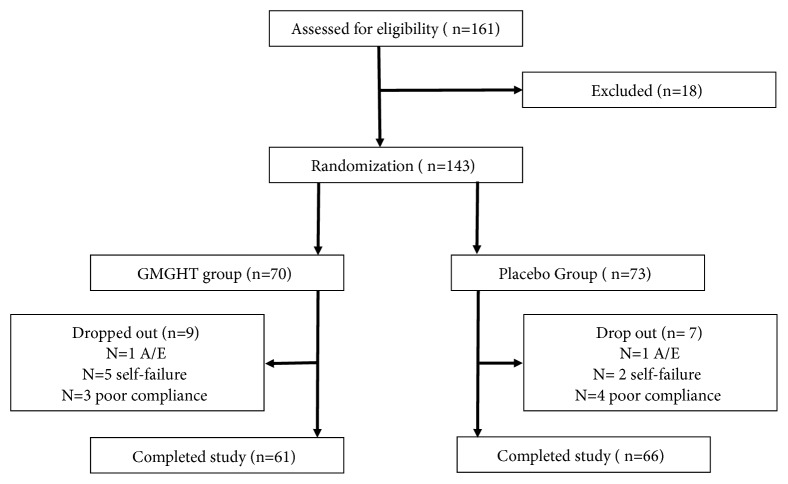
Screening, randomization, and follow-up. GMGHT, Gumiganghwal-tang; A/E, adverse event.

A total of 161 patients were recruited into this study, of whom 18 were excluded (Figure 1). The remaining 143 patients were randomized into either the GMGHT or placebo group. In the GMGHT group, 9 patients withdrew from the study due to ineffectiveness (n = 3), a transportation problem (n = 1), retraction of informed consent (n = 1), in-hospital treatment not associated with study drug but with other medical condition (car accident, n = 1) and poor compliance including insufficient drug administration, mistaken f/u (follow-up) date (n = 3). In the placebo group, 7 patients withdrew from the study due to ineffectiveness (n = 2), in-hospital treatment due to severe upper respiratory infection (n=1), and poor compliance (n = 4).

At baseline, there was no significant difference in demographic data e.g., sex, age, height, and duration of symptomatic OA between the two treatment groups except for weight and BMI. The baseline radiographic findings (Kellgren and Lawrence grade), WOMAC score, patient global assessment, patient's pain assessment, and physician's global assessment were comparable between the GMGHT and placebo group (Table 2).

**Figure 2:**
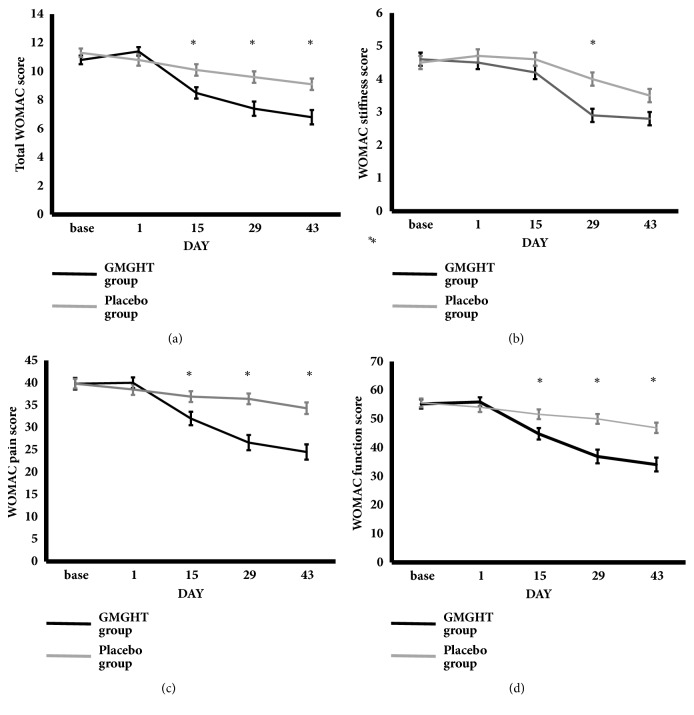
**The mean change in the Western Ontario and McMaster Universities Osteoarthritis Index (WOMAC) score**. There was significant improvement in the mean change of (a) total WOMAC, (b) WOMAC stiffness, (c) WOMAC pain, and (d) WOMAC function score from baseline in each group over the 6 weeks. *∗* indicates statistically significant results compared to baseline measurement (*p* value ≤ 0.05).

The mean total WOMAC score was significantly lower in the GMGHT group compared to the placebo group in the 2^nd^ and 4^th^ week of the study (Table 2). This difference was maintained 2 weeks later following cessation of the medication. The change in total WOMAC score from baseline also significantly improved in the GMGHT group compared to placebo group in 2^nd^, 4^th^, and 6^th^ weeks (Table 3, Figure 2).

**Figure 3:**
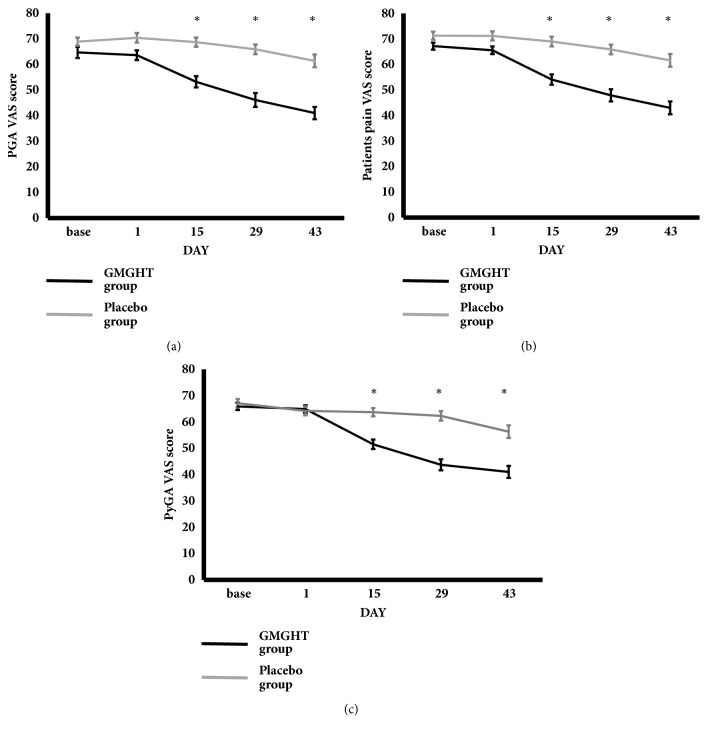
**Patient's global assessment, pain, and physician's global assessment score using the visual analogue scale**. There was significant improvement in the mean change of (a) patient's global assessment (PGA), (b) patient's pain, and (c) physician's global assessment (PhyGA) visual analogue score from baseline in each group over the 6 weeks.

The change in patient's pain assessment, patient's global assessment, and physician's global assessment from baseline significantly improved in the GMGHT group compared to the placebo group in the 2^nd^, 4^th^, and 6^th^ weeks (Table 4, Figure 3). The change in pain and function WOMAC score from baseline significantly improved in the GMGHT group compared to the placebo group in the 2^nd^, 4^th^, and 6^th^ weeks. In contrast, the change in stiffness WOMAC score from baseline was significantly different in 4^th^ week, but not in 2^nd^ week and 6^th^ week.

Adverse events were reported in a total of 30 participants: 14 patients in the GMGHT group (22.2 %) and 16 in the placebo group (23.9%, p = 0.82). There were two cases of serious adverse events, resulting in cessations of the study medication; one patient in the GMGHT group had in-hospital treatment due to a severe upper respiratory infection and another patient in the placebo group had in-hospital treatment due to a car accident. Both cases were not associated with the medication in the study. Adverse drug reactions (ADRs) were reported in a total of 28 cases: 13 cases in the GMGHT group (20.6%) and 15 cases in the placebo group (22.4%). The most common ADR was related to the gastrointestinal system: bitter taste (3 GMGHT, 4 placebo), transient dyspepsia (2 GMGHT, 4 placebo), flatulence (2 GMGHT, 3 placebo), transient epigastric pain (1 GMGHT), and transient constipation (1 placebo ). The liver function tests were elevated less than 3 times the upper limit in 3 patients (2 GMGHT, 1 placebo), and they recovered within a week without alteration of the study medications. No severe ADR was reported in either group.

## 4. Discussions

This randomized, placebo controlled study investigated the clinical efficacy and safety of GMGHT in patients with osteoarthritis of the knee. We found that administration of GMGHT resulted in significant improvement of the total WOMAC score after 4 weeks of treatment. This improvement was noticed early in the 2^nd^ week in the outcome measures of pain and function and the benefit continued for 2 weeks after cessation of the study medication.

OA is classified as noninflammatory arthritis compared to chronic inflammatory arthritis, e.g., rheumatoid arthritis or psoriasis. However, studies investigating the pathomechanism of OA suggest that inflammation is critical in OA development and progression. Although OA is a prevalent disease and our understanding of the pathomechanisms has progressed, medication for OA has not improved.

GMGHT is mixture of 9 traditional herbs and one of the most commonly used herbal prescriptions in Korea [15]. It has been used to treat the common cold, pain, and inflammatory diseases. Oral administration of GMGHT* in vivo *showed anti-inflammatory activity in 1% carrageenin and acetic acid induced edema in rats and an analgesic effect comparable to piroxicam in the writhing syndromes induced by acetic acid in mice [14].* In vitro*, GMGHT effectively inhibited the production and mRNA expression of inflammatory cytokines including tumor necrosis factor-*α* (TNF-*α*) and interleukin-6 (IL-6) on lipopolysaccharide- (LPS-) stimulated peritoneal macrophages [13]. In a dose-dependent manner, GMGHT also attenuated the mRNA and protein expression of cyclooxygenase-1 (COX-1) via suppression of nuclear factor-*κ*B (NF-*κ*B) by blocking the Rel/p65 translocation to the nucleus [13]. These data suggest GMGHT has anti-inflammatory properties and can explain why GMGHT is effective in patients with arthritis.

Pharmacological treatment for OA is largely classified as medication for slowing down the progression of OA (i.e., disease modifying OA drugs, DMOADs) or for alleviating symptoms that is further classified into symptomatic rapid acting drugs such as NSAIDs and symptomatic slow acting drugs (symptomatic slow acting drugs for OA, Sy-SADOA), which have a time to action of several weeks and a residual effect after treatment discontinuation [16, 17]. Most of Sy-SADOA, paracetamol, and topical NSAIDs are not sufficient to control pain, and oral NSAIDs are often used. Oral NSAIDs provide rapid improvement in pain, but they are associated with gastrointestinal complications and may result in deterioration [18–20]. Although selective COX-2 inhibitors showed less gastrointestinal side effects, they are associated with increased cardiovascular risk [6, 8]. As patients with OA often have at least one comorbidity, it is often difficult to prescribe these medications for OA.

In the current study, improvement of WOMAC scores was observed early in the 2^nd^ week after taking GMGHT. Furthermore, GMGHT showed rapid improvement in both pain and stiffness outcomes within 14 days of administration, which is similar to the effect of NSAIDS, which is a rapid onset symptom controlling medication for OA. Improvement in pain persisted for 14 days after discontinuation of the study drugs. These results suggest GMGHT may have a role not only as a drug for rapid onset symptomatic treatment but also as Sy-SADOAs. In contrast to NSAIDs, there were a few adverse drug reactions, including edema, and elevated serum creatinine levels. There were three patients who showed abnormal liver function tests (2 in GMGHT, 1 in placebo), but they rapidly returned to the normal range within 1 week while continuing the GMGHT medication. None of those patients took acetaminophen, a rescue medication, more than 3000mg per day which is the allowed dose in this study and within usual safe therapeutic dose. The GMGHT dose (43.9 g twice a day) in the current study is less likely to be toxic to liver; previous* in vivo* study demonstrated the no-observed-adverse-effect-level (NOAEL) for GMGHT as a dietary dose of over 2000mg/kg/day [21].* In vitro*, within concentration of 500 *μ*g/ml, GMGHT has no cytotoxic effect on hepatocytes [22]. In addition, because GMGHT inhibits cytochrome P450, especially CYP2D6 and CYPE1, it is less likely that concurrent administration of acetaminophen and GMGHT might be associated with hepatotoxicity in terms of drug-drug interaction [23].

There are several limitations in the current study. First, established medications including NSAIDs were not employed to compare the effects of GMGHT to conventional treatment. Secondly, there was a statistically significant difference in body weight and body mass index (BMI) between the placebo and treatment groups. Although obesity is a contributing factor for symptomatic knee OA, considering comparable baseline parameters (i.e., the patient's global assessment, physician's global assessment, and WOMAC scores), the difference would have had little impact on the study results. Finally, only one fixed dose of GMGHT was applied in the current study because safety and tolerability were as important as efficacy; therefore, we could not verify the effect of different doses on the efficacy and safety in OA patients. Further studies with larger sample sizes and multiple dosages are warranted.

## 5. Conclusions

This prospective, randomized, double-blinded multicenter study demonstrated that 4-week treatment with GMGHT in patients with knee OA was effective in improving pain and function and had good safety and tolerability profiles.

## Figures and Tables

**Table 1:** Composition of Gumiganghwal-tang.

Latin Name
*Angelicae Koreanae* Radix
*Peucedani japonicae* Radix
*Cnidii* Rhizoma
*Angelicae fahuricae* Radix
*Atractylodis* Rhizoma
*Scutellariae* Radix
*Rehmanniae *Radix
*Asiasari *Radix
*Glycyrrhize* Radix

**Table 2:** Demographic data and baseline characteristics.

Characteristics	GMGHT group (n= 70)	Placebo group (n= 73)	*p* value
Sex (female), n (%)	67 (95.7)	62 (84.9)	0.05
Age	58.5 (8.7)	59.7 (7.5)	0.41
Height (m)	1.57 (0.06)	1.57 (0.07)	0.77
Body weight	59.1 (6.7)	62.3 (9.3)	0.02
BMI (kg/m^2^)	23.9 (2.2)	25.2 (4.2)	0.02
Duration of symptomatic OA (months)	51.7 (54.3)	61.4 (63.3)	0.33
Radiographic findings (Kellgren and Lawrence X-ray grade )			
Grade 2, n (%)	45 (64.3)	42 (57.5)	0.43
Grade 3, n (%)	20 (28.6)	28 (38.4)	
Grade 4, n (%)	5 (7.1)	3 (4.1)	
Patient's global assessment (0-100 VAS)	65.1 (17.5)	69.2 (13.0)	0.13
Patient's pain assessment (0-100 VAS)	68.4 (11.5)	71.0 (13.6)	0.22
Physician's global assessment (0-100 VAS)	65.7 (11.8)	68.1 (13.4)	0.28
WOMAC score			
Pain	11.1 (2.3)	11.3 (3.0)	0.70
Stiffness	4.7 (1.3)	4.3 (1.4)	0.17
Function	39.6 (10.0)	40.5 (9.6)	0.55
Total	55.4 (12.7)	56.2 (12.9)	0.70

Values are presented as numbers (%) or mean (SD) unless otherwise stated.

BMI: body mass index, GMGHT: Gumiganghwal-tang, OA: osteoarthritis, SD: standard deviation, VAS: visual analog scale, WOMAC: the Western Ontario and McMaster Universities Arthritis Index.

**Table 3:** Comparisons of total WOMAC score (primary end point).

	GMGHT group (n= 61)	Placebo group (n= 66)	*p* value
Total WOMAC score			
Baseline (day =0)	55.8 (13.0)	54.1 (13.9)	0.48
2^nd^ week	45.5 (14.9)	51.6 (14.0)	0.20
4^th^ week	37.5(18.2)	50.0 (13.7)	3.07 x 10^−5^
6^th^ week	34.7 (18.1)	46.9 (14.5)	5.53 x 10^−5^
Change in total WOMAC score from baseline			
2^nd^ week	-10.3 (11.2)	-2.5 (4.6)	2.93 x 10^−6^
4^th^ week	-18.3 (16.9)	-4.1 (5.2)	2.38 x 10^−8^
6^th^ week	-21.1 (17.5)	-7.1 (8.1)	1.85 x 10^−7^

Values are presented as mean (SD) unless otherwise stated.

GMGHT: Gumiganghwal-tang, SD: standard deviation, WOMAC: the Western Ontario and McMaster Universities Arthritis Index.

**Table 4:** Comparisons of secondary efficacy.

	GMGHT group (n= 61)	Placebo group (n= 66)	*p* value
Change in patient's global assessment from baseline			
2^nd^ week	-10.4 (10.8)	-1.7 (5.8)	2.50 x 10^−7^
4^th^ week	-17.4 (19.0)	-4.5 (6.6)	3.24 x 10^−6^
6^th^ week	-22.5 (20.7)	-9.0 (12.8)	3.04 x 10^−5^
Change in patient's pain assessment from baseline			
2^nd^ week	-11.1 (11.0)	-2.2 (5,2)	1.32 x 10^−7^
4^th^ week	-17.5 (19.7)	-5.4 (6.6)	2.07 x 10^−5^
6^th^ week	-22.4 (21.3)	-9.6 (14.4)	1.62 x 10^−4^
Change in physician's global assessment from baseline			
2^nd^ week	-13.3 (12.0)	-0.5 (6.1)	4.67 x 10^−11^
4^th^ week	-21.0 (19.4)	-1.8 (8.3)	3.89 x 10^−10^
6^th^ week	-23.8 (20.9)	-7.9 (16.9)	6.57 x 10^−6^
Change in pain WOMAC score from baseline			
2^nd^ week	-2.8 (2.6)	-0.7 (1.8)	1.45 x 10^−6^
4^th^ week	-3.9 (4.0)	-1.2 (2.0)	8.12 x 10^−6^
6^th^ week	-4.5 (4.0)	-1.7 (2.4)	6.82 x 10^−6^
Change in stiffness WOMAC score from baseline			
2^nd^ week	-0.2 (1.4)	-0.1 (1.3)	0.75
4^th^ week	-1.6 (1.6)	-0.7 (1.3)	1.27 x 10^−3^
6^th^ week	-1.6 (1.6)	-1.3 (1.7)	0.22
Change in function WOMAC score from baseline			
2^nd^ week	-7.3 (8.6)	-1.7 (3.0)	7.49 x 10^−6^
4^th^ week	-12.8 (12.2)	-2.2 (3.3)	7.16 x 10^−9^
6^th^ week	-15.0 (12.8)	-4.2 (5.3)	3.26 x 10^−8^

Values are presented as mean (SD) unless otherwise stated.

GMGHT: Gumiganghwal-tang, SD: standard deviation, WOMAC: the Western Ontario and McMaster Universities Arthritis Index.

## Data Availability

The data used to support the findings of this study are available from the corresponding author upon request.

## Abbreviations

ACR: American College of Rheumatology
ADR: Adverse drug reaction
BMI: Body mass index
COX 2: Cyclooxygenase
DAMPs: Danger-associated molecular patterns
DMOADs: Disease modifying osteoarthritis drugs
GMGHT: Gumiganghwal-tang
IL-6: Interleukin-6
LPS: Lipopolysaccharide
NF-*κ*B: Nuclear factor-*κ*B
NSAIDs: Nonsteroidal anti-inflammatory drugs
OA: Osteoarthritis
PAMPs: Pathogen-associated molecular patterns
Sy-SADOA: Symptomatic slow acting drugs for osteoarthritis
TNF-*α*: Tumor necrosis factor-*α*
TXA 2: Thromboxane A2
VAS: Visual analogue scale
WOMAC: Western Ontario and McMaster Universities Osteoarthritis Index.
